# Web-Based Interventions for Behavior Change and Self-Management: Potential, Pitfalls, and Progress

**DOI:** 10.2196/med20.1741

**Published:** 2012-08-14

**Authors:** Elizabeth Murray

**Affiliations:** ^1^e-Health UnitResearch Department of Primary Care and Population HealthUniversity College LondonLondonUnited Kingdom

**Keywords:** Internet, self-care, eHealth, health behavior

## Abstract

The potential advantages of using the Internet to deliver self-care and behavior-change programs are well recognized. An aging population combined with the increasing prevalence of long-term conditions and more effective medical interventions place financial strain on all health care systems. Web-based interventions have the potential to combine the tailored approach of face-to-face interventions with the scalability of public health interventions that have low marginal costs per additional user. From a patient perspective, Web-based interventions can be highly attractive because they are convenient, easily accessible, and can maintain anonymity/privacy. Recognition of this potential has led to research in developing and evaluating Web-based interventions for self-management of long-term conditions and behavior change. Numerous systematic reviews have confirmed the effectiveness of some Web-based interventions, but a number of unanswered questions still remain. This paper reviews the progress made in developing and evaluating Web-based interventions and considers three challenging areas: equity, effectiveness, and implementation. The impact of Web-based interventions on health inequalities remains unclear. Although some have argued that such interventions can increase access to underserved communities, there is evidence to suggest that reliance on Web-based interventions may exacerbate health inequalities by excluding those on the “wrong” side of the digital divide. Although most systematic reviews have found a positive effect on outcomes of interest, effect sizes tend to be small and not all interventions are successful. Further work is needed to determine why some interventions work and others do not. This includes considering the “active ingredients” or mechanism of action of these complex interventions and the context in which they are used. Are there certain demographic, psychological, or clinical factors that promote or inhibit success? Are some behaviors or some clinical problems more amenable to change by computer-based interventions? Equally problematic is the issue of implementation and integration of such programs into routine clinical practice. Many eHealth projects end when the research is concluded and fail to become part of mainstream clinical care. One way of addressing these challenges is to apply the Medical Research Council framework for developing, evaluating, and implementing complex interventions. This includes having a strong theoretical foundation, developing a proposed mechanism or pathway of action, ensuring that the evaluation adequately reflects this proposed pathway, and considering implementation from the beginning of the development process.

## Background

The challenges facing health care systems in the 21^st ^century include an aging population, increasing prevalence of long-term conditions, improved survival rates because of new health technologies, and rising consumer expectations of health care. All of these combine to put ever-increasing pressure on available health care resources [1]. Although each country is pursuing individual solutions to these challenges, some common approaches are apparent, such as the use of information and communication technology (ICT) or eHealth. The use of ICT is expected to lead to improvements in health care quality (eg, through better communication) and efficiency (eg, through reduced duplication of investigations) [2,3]. Similarly, there is an increasing emphasis on promoting self-care or self-management by patients, both for patients with long-term conditions and a more general promotion of healthy behaviors [4].

The importance of encouraging healthy behaviors is clear. Five behaviors—poor diet, lack of exercise, smoking, excessive alcohol consumption, and unprotected sex—account for approximately 39% of deaths in the United States [5]. There would be a substantial improvement in public health if large numbers of people were to stop (or not start) smoking, drink in moderation, practice safer sex, eat healthily, and increase their level of physical activity [4,6,7]. There is also a need to promote self-management for patients with long-term conditions, such as diabetes, heart disease, or arthritis. Effective self-management programs have been shown to reduce health care costs and improve quality of life across a range of conditions [8-10].

These two policy imperatives, increasing the use of ICT and enhancing people’s ability to self-care and adopt healthy behaviors, intersect in the field of Web-based interventions. The aim of this paper is to review the potential benefits of Web-based interventions, consider issues that require further attention before these potential benefits can be realized, and suggest ways of moving forward.

## Definitions

### Web-Based Interventions

As Barak et al [11] have described, there has been a lack of clarity and consistency in the field of Internet-supported therapeutic interventions. This paper focuses on Barak et al’s “Web-based interventions” defined as:

*“...a primarily self-guided intervention programme that is executed by means of a prescriptive online programme operated through a website and used by consumers seeking health- and mental-health related assistance. The intervention programme itself attempts to create positive change and or improve/enhance knowledge, awareness, and understanding via the provision of sound health-related material and use of interactive Web-based components.”*

The key components of such interventions include program content, use of multimedia, interactive online activities, and guidance or supportive feedback [11]. Web-based interventions have been developed for three main clinical areas: self-management of long-term conditions (eg, diabetes, heart disease, arthritis, and asthma), health promotion (eg, smoking cessation, alcohol reduction, sexual health, diet, and exercise), and mental health (eg, depression and anxiety).

### Self-management

Self-management is a complex concept. In their seminal work, *Unending Work and Care: Managing Chronic Illness at Home, *Corbin and Strauss [12] identified three tasks required for self-management: medical management; emotional management, and role management. Medical management receives the most attention from health professionals and designers of many Web-based interventions. It includes remembering to take medications regularly, managing interactions with health professionals, and adopting healthy behaviors such as eating healthily, exercising more, or stopping smoking. From a patient perspective, the other two tasks are just as important and just as challenging. Emotional management refers to the work required for individuals to come to terms with the very strong negative emotions (eg, guilt, shame, anger, and despair) that accompany a long-term condition. Role management is the work required to adapt to the changes in social roles and relationships (eg, at work, within the family, or among friends) caused by the long-term condition [12].

## Potential

The potential benefits of eHealth interventions for reach, accessibility, effectiveness, and cost-effectiveness have been described elsewhere [13,14]. The Internet is now widely available in the developed world, with more than 90% penetration in Sweden, approximately 80% penetration in the United Kingdom, Australia, and the United States, and approximately 60% in Europe as a whole [15]. An almost unlimited amount of information can be stored on the Web, yet it can be presented in an accessible and comprehensible format, in bite-size chunks, using video, graphics, and audio, and is available at the moment of need. Interactivity is a major advantage of the Internet, both for users interacting with websites and entering personal information that allows the site to tailor the information or services offered to that specific user, and for users interacting with one another through the Web. These two forms of interactivity can be used to provide formal behavior change support, decision support, and peer support. Delivering behavior change interventions over the Internet is appealing because this could combine the scalability of public health interventions with the effectiveness of personalized, individually tailored interventions.

Finally, because the main costs of Internet interventions tend to be incurred during the development phase and the marginal cost per additional user tends to be low, they have the potential to be highly cost-effective, particularly when used by large numbers of people [16,17].

## Pitfalls

Many of these potential benefits have yet to be realized. This section outlines current difficulties in three areas (ie, equity, effectiveness, and implementation) that require further attention before the potential of Web-based interventions can be realized.

### Equity

Web-based interventions could have two opposing impacts on health inequalities. Some have argued that Web-based interventions could help reduce health inequalities by opening up access to health information that was previously only accessible by health professionals. Reducing information asymmetry may help reduce the power imbalance between health professionals and patients, thus enabling patients to play a more active role in their health care [18,19]. Moreover, the use of multimedia (eg, video, audio, and graphics) allows complex information to be presented in a simple format comprehensible even to those with low literacy skills. Web-based interventions may also reduce health inequalities by improving access to services. One example is the provision of mental health services in Australia. Patients in sparsely populated rural areas find it difficult to access therapists trained in cognitive behavioral therapy (CBT); delivery of online CBT, with or without email support from a therapist, enables these rural patients access to this therapy [20].

An alternative view is that widespread use of Web-based interventions will widen health inequalities between those on either side of the “digital divide” [21]. Those with access to the Internet will benefit from an increasing array of services, whereas those without will find themselves increasingly disadvantaged by not being able to access health information or services [22]. Moreover, access is necessary—but not sufficient—for benefiting from Web-based interventions. Once people have gained access to such interventions, they have to go through a complex process of reading the information or other content, interpreting and making sense of it, and applying it to their personal circumstances. This ability to use and benefit from a Web-based intervention once provided with access can be thought of as “accessibility.” Accessibility could be affected by many factors. One factor will be user levels of literacy and health literacy, in which health literacy is defined as the ability of individuals to gain access to, understand, and use information for health [23]. Other factors are likely to include internal and external constraints that may prevent users from acting on information. External constraints that may prevent health-promoting activities include poor housing, poor working conditions, and poverty [24]. Internal, or psychological, constraints (eg, low self-efficacy or an external locus of control) may also play a part.

There are data to support both arguments. Some researchers have undertaken impressive work, demonstrating that Internet interventions can be used by and can benefit severely disadvantaged groups, including homeless drug users [25], single teenage mothers [26], and vulnerable elderly people [27]. In all of these studies, attention was paid both to providing access (eg, hardware and training in use of the system) and to accessibility (eg, ensuring the content and presentation were relevant and meaningful to the target audience). These studies demonstrate that if the problems of access and accessibility are both addressed, Web-based interventions have the potential to reach and to benefit socioeconomically deprived people.

Unfortunately, outside of these carefully designed and executed studies, there is evidence that Web-based interventions tend to be used by more advantaged groups. Looking first at the relatively simple question of access to the Internet, it is clear that there are considerable inequities in access, both between and within countries. In contrast to the high levels of Internet penetration seen in the United States, Australia, and Europe, Internet penetration in Africa was estimated at 11%, 24% in Asia, and 36% in Latin America and the Caribbean in 2011 [15]. Even in countries where there is a relatively high level of access, such as the United Kingdom, there are access inequalities within the country. Access to the Internet increases with income and education. In 2011, Internet access among people with household incomes of £40,000 or more was 99%, whereas it was only 43% among people with household incomes of £12,500 or less (half the national median income). Similarly, among people with a university degree, access was 95%, whereas access was only 31% for those people who left school without graduating. Of greater concern, older people and people with disabilities or long-term illnesses are less likely to have Internet access. In 2011, Internet access among those aged 65 or over was <40%, and among those with health problems or disabilities that limited the kind or amount of work able to be done, access was 41% [28]. In other words, those with the most need have the least access. Our own research on Web-based interventions supports these concerns about equity, with disproportionately high numbers of users having a university degree [29,30].

## Effectiveness

Barak et al’s definition of Web-based interventions posits an intention to create positive change across a range of outcomes. Apart from knowledge or understanding, these outcomes are undefined, but the conceptual framework of self-management developed by Corbin and Strauss implies that relevant outcomes are likely to include cognitive, behavioral, and emotional outcomes. Cognitive outcomes include knowledge or understanding, intention (eg, to adopt a particular healthy behavior), and self-efficacy (eg, a belief in one’s capacity to undertake an intended task or behaviors). Behavioral outcomes include changes in diet, physical activity, smoking cessation, moderating alcohol consumption, and managing medicines safely and effectively. Emotional outcomes that may be targeted include strong negative emotions such as anger, guilt, shame, depression, and anxiety. Improvements in emotional and behavioral outcomes are likely to lead to improvements in clinical outcomes and/or well-being [31]. This next section explores the data on the effectiveness of Web-based interventions for these three outcome categories followed by a discussion of how such interventions achieve their effects.

### Cognitive Outcomes

Web-based interventions can improve knowledge. Computer-assisted learning (CAL) has been used as an educational tool since the 1960s [32] and there are ample data demonstrating its efficacy [33]. In the health field, there have been a number of systematic reviews and meta-analyses that have demonstrated improvements in knowledge in users of Web-based interventions [31,34]. There are also data suggesting that some, but not all, Web-based interventions have a positive impact on mediators of behavior change such as intention and self-efficacy [34].

### Behavioral Outcomes

From a clinical perspective, there are two main populations to target for behavior change: currently healthy people who are engaging in unhealthy behaviors likely to result in future physical health problems (health promotion or primary prevention) and people who are currently unwell and need to change their health behaviors to prevent further deterioration of their health status (self-management of long-term conditions or secondary prevention). The behaviors of interest tend to be the same across both populations (ie, healthy eating, physical activity, smoking cessation, moderating alcohol consumption, and practicing safer sex), but the levels of motivation may differ.

From a public health perspective, a small change across a large population can have a significant impact on public health [35]. Hence, the positive findings of that numerous systematic reviews and meta-analyses of the effects of Web-based interventions for specific behaviors including smoking cessation [36], reducing alcohol consumption [37], safer sexual behaviors [34], and increasing physical activity [38] are reassuring even if the effect sizes tend to be small. Changing dietary behaviors appears to be more challenging, with a recent systematic review for the UK Health Technology Agency finding little or no impact of adaptive e-learning technologies across a wide range of dietary behaviors and no impact on weight loss or body mass index [39]. Systematic reviews that have looked across a range of behaviors have demonstrated similar findings [40,41].

Although these findings are reassuring at a population level and may support the use of Web-based interventions for health promotion, the data on Web-based interventions for people with long-term conditions is less positive. It can be postulated that the motivation of people with a long-term condition differs from those without a diagnosis of a health problem because the need for change is both more immediate and more personal. For this population, the goal of behavior change tends not to be an end in itself, but rather as a step toward improved clinical outcomes [31]. Although small changes in behavioral outcomes have been demonstrated, these have not converted into improved clinical outcomes [31].

An alternative approach has been the use of computerized cognitive behavioral therapy (CCBT), which was first developed for use with people experiencing mental health problems such as depression and anxiety [42,43]. The effectiveness of CCBT for mental health problems is discussed subsequently. More recently, interventions offering CCBT have been developed for a range of somatic problems such as tinnitus, insomnia, chronic pain, and headache. Although initial results are promising, further work is needed because many of the trials have been relatively small [44-47].

### Emotional Outcomes

There has been a great deal of work on Web-based interventions for mental health. Interventions based on CCBT have been shown to be acceptable, effective, and cost-effective across a range of mental health problems including mild to moderate depression, anxiety, obsessive-compulsive disorder, and phobias [48-54].

Web-based interventions not based on CCBT that are aimed at improving emotional outcomes have been less successful. Two main alternative approaches have been used: Web-based social networks and provision of personal stories. Personal stories are narratives from people experiencing similar health problems to the user. The underlying concept is that the user will obtain emotional and informational support by reading about “someone like them” who has had similar experiences. Perhaps the most well-known example of this approach is Health Talk Online (www.healthtalkonline.org), previously known as the Dictionary of Patient Experiences (DIPEX). Health Talk Online currently provides access to more than 2000 patient narratives about approximately 60 different health problems. Qualitative data suggest that users find such “personal experiences” helpful in reaching decisions [55], but there are less data available about whether and how such narratives impact emotional or clinical outcomes [56].

Online social networks can be incorporated into Web-based interventions in the form of chat rooms, bulletin boards, or forums. Such social networks have a strong appeal to some users, both because of the ability to interact with other people going through similar experiences and because of their availability at all hours [57]. Unfortunately, the research to date has failed to identify evidence of benefit (or harm) to users of online peer-support services. Authors of systematic reviews in this field have highlighted the paucity of comparative data, making it difficult to draw any robust conclusions about the impact of such interventions [58-60].

### Mechanism of Action

Systematic reviews of Web-based interventions make it apparent that some interventions work better than others do. This may be because of differences in target populations, target behaviors, target outcomes, or differences in the content or delivery of the interventions themselves. To date, it has not been possible to untangle this, although it is possible to draw some tentative conclusions.

Some of the potential differences between healthy populations and those with a long-term condition have been mentioned in the preceding sections, and it seems likely that unsupported use of a Web-based intervention requires a fairly high degree of motivation and critical health literacy [61] on the part of the user. Among target behaviors, weight loss seems particularly difficult to achieve with Web-based interventions [39].

Focusing on content and delivery of interventions suggests that those with a theoretical foundation are more likely to be effective than those without [41,62]. Interventions offering CCBT appear to have a good chance of being effective across a range of physical and mental health problems known to respond to face-to-face CBT. Behavior-change interventions that do not use CBT techniques can also be effective. A recent large systematic review and meta-analysis of Web-based behavior-change interventions aimed to identify why some interventions worked and some did not. The review included approximately 85 studies covering more than 45,000 participants. The authors applied a newly developed taxonomy of behavior-change techniques to interventions in the included studies. From this analysis, they were able to conclude that more extensive use of theory (particularly the Theory of Planned Behavior) and certain behavior-change techniques (eg, stress management, goal setting, and action planning) were associated with larger effect sizes [41]. A similar methodological approach, but focusing on the use of persuasive features and mechanisms embedded in Web-based interventions, found considerable use of persuasive techniques. However, the authors were unable to conclude whether specific techniques were associated with effectiveness [63].

Equally important as considering the “active components” of the intervention is consideration of what constitutes an “effective dose” (ie, what level of engagement is needed for users to benefit from use of the intervention, to what extent does the “effective dose” vary between users, and what characteristics of users are likely to influence the “dose” that is needed). Attrition from Web-based interventions is a phenomenon that has been observed frequently [64] that may undermine effectiveness. Adherence to any specified intervention may be related to characteristics of the intervention, characteristics of the user, or characteristics of the condition addressed by the intervention. Characteristics of the intervention that may improve adherence to the intervention include a strong theoretical foundation [41], perceived personal relevance to the user [65,66], perceived effectiveness [67,68], tailoring [69,70], persuasive technologies [71], credibility [72,73], social networking [74,75], and regular “push factors” including human support [76-78] and/or periodic prompts (eg, by email or telephone) [79]. Data on user characteristics are confusing and contradictory. Although many researchers have found that women, older people, and well-educated people are all more likely to demonstrate adherence to Web-based interventions than males, younger people, and less-educated people [65,66,68,69], others have found no association between adherence and age, gender, or education level [80,81]. Clearly, further work is needed in this area.

### Cost-Effectiveness and Implementation

The potential cost-effectiveness of Internet interventions is highly appealing, but an intervention has to be effective before it can be cost-effective. Even when an intervention is effective, the main costs tend to be incurred during development, with relatively low marginal costs per additional user, so that any cost-benefit relies on achieving large numbers of users [17]. Although there are some interventions that have been used by very large numbers of people [82,83], many interventions have not been widely disseminated. One way of ensuring an intervention is used by large numbers of people is to integrate it into routine health services, but this has been rarely done to date. Implementation of eHealth has proved problematic in most countries, with numerous reports of delay, budget overspends, and occasional severely negative impacts on the quality and effectiveness of care [84,85]. These difficulties have continued despite the considerable literature available about implementing eHealth systems and the growing awareness of the importance of a sociotechnical approach [86,87].

There is also a paucity of data on actual cost-effectiveness of Internet interventions [17,34], making it difficult to determine their actual, versus potential, cost-benefits. Fortunately, this appears to be changing as newer studies include cost-effectiveness data [88,89].

## Progress

The previous section described areas that need further attention before Web-based interventions can achieve their full potential. This section suggests ways to move forward within these areas of research.

### Apply the Medical Research Council Framework

Most Web-based interventions are complex interventions (ie, interventions that are made up of a number of components that may act independently or interdependently). Therefore, the Medical Research Council (MRC) framework for developing, evaluating, and implementing complex interventions offers an appropriate framework for research on Web-based interventions. This framework was first published in 2000 [90] and revised in 2008 [91]. It has been highly influential, with many additional publications on applying, extending, and revising the framework [92-94]. It suggests a phased approach, with early work (Phases 0 to 2) consisting of systematic literature reviews to identify what is already known, theoretical work to establish an appropriate theoretical foundation, modeling studies to establish potential rate-limiting steps and population impact, qualitative studies to determine acceptability and feasibility, studies to confirm (or deny) the proposed pathway of action and identify appropriate intermediary outcomes, and pilot studies to optimize both the intervention and the trial parameters. These phases and studies are iterative, with development of the intervention and the evaluation methods proceeding in tandem [91,92]. Only when both the intervention and the trial parameters have been optimized and the mechanism of action has been well defined, should researchers proceed to a Phase 3 randomized controlled trial to establish the effectiveness of the intervention. Phase 4 (implementation) studies are needed both to establish how best to implement the intervention into routine practice and to determine whether the benefits shown in the trial manifest outside the trial environment. Phase 4 studies can also identify unintended adverse effects [90,91].

### Apply Theory to the Development of Web-Based Interventions

The MRC framework advises the use of a theoretical framework for development of the intervention, and this advice is supported by the empirical literature which demonstrates that theoretically informed Web-based interventions are more likely to be effective than those without a theoretical foundation. Theories offer us generalizable frameworks that can apply across different settings and individuals, the opportunity for incremental accumulation of knowledge, and an explicit framework for analysis [95]. Researchers interested in developing a Web-based behavior-change intervention have a wide range of psychological theories from which to choose. Once an appropriate theory is selected, researchers must identify the key constructs of the theory and consider how the intervention will act on these constructs based on the available data to support the use of that theory in the selected population for the target behavioral change. A simple example would be an intervention based on social cognitive theory (SCT). Key constructs of SCT are self-efficacy (an individual’s confidence in his or her ability to carry out a behavior) and outcome expectations (beliefs about the outcomes that are likely to result from a particular behavior). Hence, an intervention based on SCT would target users self-efficacy (eg, by breaking the behavior down into small, manageable steps or providing case histories of people who had achieved this behavior with details of how they managed it) and outcome expectancies (eg, by providing information about the benefits of change).

Researchers should publish detailed descriptions of the interventions they have developed, along with an explanation of the chosen theoretical framework and how it was operationalized for the active ingredients and the proposed pathway of action [92,96]. Such publications will allow other researchers to learn from their experience and allow subsequent researchers to look across a range of interventions and identify common approaches associated with effectiveness.

### Apply Theory to the Evaluation of the Web-Based Intervention

The MRC framework emphasizes the need for formative and pilot studies during the development of a complex intervention. These early phase studies aim to optimize both the intervention and the evaluation, with a view to ensuring that a final Phase 3 trial will provide a definitive answer. Early phase studies should reflect the theoretical framework used for development of the intervention. Thus if an intervention has been designed to cause changes in self-efficacy and outcome expectancies, it is important that the evaluation determines whether changes in these proximal outcomes occur as well as whether the target behavior changes. This provides empirical support for the proposed “pathway of action” of the intervention. Mediational analysis can be used to explore whether these proximal outcomes mediate the final outcome.

Phase 2 studies should also address the issue of defining and achieving an “effective dose” of the intervention. Kraft [97] has advised researchers to consider the use of an intervention as the first behavior change to target. He advocates “tunnelling,” or highly tailored pre-determined routes through the intervention, coupled with numerous proactive contacts by the intervention to the user delivered through short message service (SMS), email, or automated phone calls [98,99]. An alternative approach used in the mental health field is “facilitated access” in which use of the intervention is monitored and supported by a health care worker or therapist. Such support may be associated with improved outcomes for some conditions [51,100,101]. Eysenbach [64] has argued that researchers need to document and publish the content of interventions just as they should document both the planned and actual level of engagement by participants with interventions under evaluation.

### Apply Theory to the Implementation of Web-Based Interventions

The eHealth researcher’s responsibility is not limited to developing and evaluating effective Web-based interventions. As we have seen previously, the main cost-benefits of such interventions tend to be achieved with large numbers of users. Achieving large numbers of users often requires implementing and integrating interventions into routine health care, but this has proved difficult to date. Difficulties in eHealth implementation are international phenomena, with similar problems being widely reported [102-105]. Application of theory is likely to enhance the effectiveness and success of implementation initiatives.

The sociotechnical systems approach argues that it is the interaction between social and technical systems within an organization or context that is important for successful functioning. The recognition of the importance of both social and technical factors is reflected in a number of sociological theories pertinent to eHealth implementation, including the influential actor-network theory (ANT). ANT posits that both people and objects or technologies are actors in a social network and that disruption to any of the actors disrupts the network. Important contributions have been made to understanding the role of attitudes [106], and social transmission of innovations between [107] or interactions within [108,109] actor-networks. Gidden’s Structuration Theory reconfigures the relationship between individual agency and social structures by considering them as a duality rather than two separate entities [110] and has been applied extensively to information technology and eHealth [111].

A theory that specifically focuses on implementation, integration, and embedding of new practices is normalization process theory (NPT). The precursor to NPT, normalization process model (NPM), originated from a large body of work on eHealth and was developed to explain the observation that many eHealth initiatives failed to embed into routine practice [112,113]. NPT focuses on the “work” that health professionals and patients need to do for a new practice or technology to be implemented and become integrated and embedded into routine practice (ie, normalized). Normalization is important for sustainability. Once a new practice has become normalized or taken for granted, it is so embedded into routine practice that it requires relatively little effort to sustain its use [114].

NPT has been used to explain the success or failure of a range of eHealth implementations [115-117] and there is empirical support for its use as an explanatory model [118]. The current focus of NPT research is to determine whether it can be used prospectively, to move beyond explaining success or failure of an implementation toward planning successful implementations. To this end, the NPT toolkit (www.normalizationprocess.org) has been developed to help researchers consider implementation issues from the early stages of developing and evaluating complex interventions [119]. A similar toolkit, the eHealth Implementation Toolkit (e-HIT), is aimed specifically at eHealth interventions and has been developed for use by managers and other staff charged with implementing eHealth initiatives (ie, implementers) [120-122]. Research is underway to see if these tools have predictive utility. In the meantime, eHealth researchers should consider implementation issues from the beginning of the development and evaluation of a new intervention, including identifying and applying an appropriate theoretical approach to the issues of implementation.

## Conclusions

The eHealth field is developing quickly, but there are still many challenges to face before it can reach its full potential. This paper has used the example of Web-based interventions to focus on three particular challenges: equity, effectiveness, and implementation. It has argued that the application of the MRC framework for development, evaluation, and implementation of complex interventions, together with a greater use of theory could help address these challenges. Specific recommendations include better descriptions of Web-based interventions in the published literature, including clear descriptions of the theoretical approach used, how this was operationalized, and what the developers consider the likely pathway of action and the active components of the intervention. This will facilitate improved research designs to evaluate interventions because important mediators or proximal outcomes will be clearly specified. Consideration of implementation issues needs to start at the point of development of a new intervention. Use of an appropriate theoretical framework (eg, NPT and its associated tool kit) may help researchers work through implementation issues in a structured fashion.

## Abbreviations

ANT: actor–network theory
CAL: computer-assisted learning
CBT: cognitive behavioral therapy
CCBT: computerized cognitive behavioral therapy
DIPEX: Dictionary of Patient Experiences
e-HIT: eHealth Implementation Toolkit
ICT: information and communication technology
MRC: Medical Research Council
NPM: normalization process model
NPT: normalization process theory
SCT: social cognitive theory
SMS: short message service
